# Effect of *Ducrosia anethifolia* methanol extract against methicillin resistant *Staphylococcus aureus* and *Pseudomonas aeruginosa* biofilms on excision wound in diabetic mice

**DOI:** 10.3389/fcimb.2024.1386483

**Published:** 2024-05-02

**Authors:** Yasir Almuhanna

**Affiliations:** Department of Clinical Laboratory Science, College of Applied Medical Sciences, Shaqra University, Shaqra, Saudi Arabia

**Keywords:** LCMS analysis, cytotoxicity, epithelization, HaCaT (human keratinocyte), skin irritation

## Abstract

**Background:**

*Ducrosia anethifolia* is an aromatic desert plant used in Saudi folk medicine to treat skin infections. It is widely found in Middle Eastern countries.

**Methods:**

A methanolic extract of the plant was prepared, and its phytoconstituents were determined using LC-MS. *In-vitro* and *in-vivo* antibacterial and antibiofilm activities of the methanolic extract were evaluated against multidrug-resistant bacteria. The cytotoxic effect was assessed using HaCaT cell lines *in-vitro*. Diabetic mice were used to study the *in-vivo* antibiofilm and wound healing activity using the excision wound method.

**Results:**

More than 50 phytoconstituents were found in the extract after LC-MS analysis. The extract exhibited antibacterial activity against both the tested pathogens. The extract was free of irritant effects on mice skin, and no cytotoxicity was observed on HaCaT cells with an IC_50_ value of 1381 µg/ml. The ointment formulation of the extract increased the healing of diabetic wounds. The microbial load of both pathogens in the wounded tissue was also reduced after the treatment. The extract was more effective against methicillin-resistant *Staphylococcus aureus* (MRSA) than MDR-*P. aeruginosa* in both *in vitro* and *in vivo* experiments. Further, skin regeneration was also observed in histological studies.

**Conclusions:**

The results showed that *D. anethifolia* methanol extract supports wound healing in infected wounds in diabetic mice through antibacterial, antibiofilm, and wound healing activities.

## 1 Introduction

Medicinal plants with potent antimicrobial effects are used traditionally in Middle Eastern countries (Ullah et al., 2020). Earlier reports show that Saudi medicinal plants have good antibacterial and anti-inflammatory effects and are widely used in traditional medicine to treat infections and wounds (Shahat et al., 2017; El-Seedi et al., 2022). However, these plants have not been explored for their antimicrobial effects, especially against multidrug-resistant pathogenic infections and biofilm formation.

One of the plants commonly used in the Kingdom of Saudi Arabia for wound treatment is the leaves of *Ducrosia anethifolia* Bois, belonging to the family- Apiaceae (Flora of Saudi Arabia by Ahmed Mohammed Migahid | Open Library). The plant is also used to treat skin infections in several other countries, including Afghanistan, Pakistan, Iran, Iraq, and other Arabian countries (Mottaghipisheh et al., 2020). It is locally called ‘*Al-Haza*’ in Arabic and is a desert plant that grows in Saudi Arabia’s volcanic cinders. This plant is a biennial herb and is drought-resistant. Earlier reports show that the plant possesses different pharmacological effects. Some of the activities reported include anti-diabetic and antiulcer effects (Unissa Syed et al., 2022), analgesic, central nervous system depressant actions such as anti-anxiety, sedative, and anti-depressant effects (Abbaszadeh et al., 2019), carminative, relief of colic pain and as a flavoring agent (Mottaghipisheh et al., 2020). Further there are reports on phytoconstituents present in *Ducrosia anethifolia* showing antibacterial activity against MRSA (Mahboubi et al., 2014).

Infections in wounds are prevalent due to exposure of wounded tissue to bacteria. The infectious organism usually forms a biofilm over the wounded tissue within 24 hours to escape the attack from the patient’s immune system and attenuate the effect of antimicrobial agents. Biofilms are bacteria aggregates embedded in a barrier consisting of sugars and proteins (Flemming et al., 2016). These are considered the single most common cause of delay in wound healing, and they delay the wound healing process through an inappropriate inflammatory response that damages the wounded tissue (Darvishi et al., 2022). Hence, agents used in the treatment of wounds should not only possess antimicrobial effects but should effectively prevent and eradicate biofilm formation over the wounded tissues (Thapa et al., 2023). The two most common pathogens causing skin infections include *Methicillin-resistant Staphylococcus aureus* (MRSA) and *multi-drug-resistant- Pseudomonas aeruginosa* (MDR-*P. aeruginosa*). MRSA is associated with community-acquired skin and soft tissue infections as well as nosocomial infections (Odell, 2010; Pannewick et al., 2021). Furthermore, there are earlier reports on the effect of essential oils and decanal, a component of *D. anethifolia* against MRSA, wherein it was shown that more than one phytoconstituent of *D. anethifolia* is responsible for its antimicrobial effect (Mahboubi and Feizabadi, 2009). MDR-*P. aeruginosa* is one of the most common infective organisms for skin and soft tissue infections (Wu et al., 2011). An earlier study indicates that hydroalcoholic extract of *D. anethifolia* from Jordan inhibits *P. aeruginosa in-vitro* (Nawash et al., 2013).

Many plant extracts have been reported for antibiofilm effects. Traditional plants from Pakistan, such as *Bergenia ciliata, Clematis grata*, and *Clematis viticella*, are reported to inhibit *P. aeruginosa* biofilms (Alam et al., 2020). Similarly, African medical plants such as *Alchornea laxiflora, Ficus exasperata, Morinda lucida, Jatropha gossypiifolia, Ocimum gratissimum*, and *Acalypha wilkesiana* were shown to inhibit biofilm formation by various pathogens (Olawuwo et al., 2022). Medical plants from Argentina, such as *Lycium chilense* and *Schinus fasciculatus*, have also been reported for anti-biofilm effects against various pathogens (Romero et al., 2016). Most of the studies on the antibiofilm activities of plant products have been carried out using *in-vitro* methods that do not provide sufficient evidence that these plants will be effective antibiofilm agents *in vivo* (Lu et al., 2021; Younis et al., 2021; Priyanto et al., 2022). Furthermore, phytoconstituents present in some of the extracts are not known (Alam et al., 2020; Zammuto et al., 2022). The active chemical constituents present in the plant extracts help in the development of novel molecules (Harikrishnan et al., 2021; Oselusi et al., 2021).

The present study evaluated the unexplored antimicrobial, antibiofilm, and wound healing of *Ducrosia anethifolia* to confirm its traditional use as an anti-infective agent on skin wounds in diabetic animals. Furthermore, an attempt was made to identify phytoconstituents present in the methanolic extract of the leaves through liquid chromatography-mass spectrometry (LC-MS) analysis that may help in the identification of lead molecules. The skin irritant effect of the prepared extract formulation was evaluated on the mouse skin *in-vivo* and on human keratinocytes (HaCaT) *in-vitro* to determine the safety.

## 2 Materials and methods

### 2.1 Chemicals

Chemicals of analytical grade purchased from local chemical suppliers were used.

### 2.2 Animals

Swiss albino mice (27 to 30 g) maintained under a controlled environment were utilized. The experimental procedure was approved by the Ethical Research Committee of Shaqra University (No. ERC SU_20220066).

### 2.3 Extract preparation and phytochemical analysis

The herb was collected in August 2022, followed by authentication in the institute by a botanist. A specimen of the herb (No. SU/CAMS/09/2022) is maintained in the institute as a reference. The plant was shade-dried, coarsely powdered, subjected to Soxhlet extraction using methanol, and dried in a rotavapor (Mukherjee, 2019). The extract yield obtained was 26.34% w/w.

The extract was injected into the waters LC instrument (XEVO-TQD#QCA1232) having a C_18_ column (250 mm X 2.1 mm, 2.6 µm). The flow rate was maintained at 0.2 ml/min, and detection was carried out at 280 nm. Acetonitrile and ammonium formate buffer were used as solvents with gradient conditions as reported by Al-Ghanayem et al (Al-Ghanayem et al., 2022a). The spectra were recorded at ionization modes from m/z 150 to 2000.

### 2.4 Antibacterial activity and antibiofilm activity *in-vitro*

Antibacterial effects of the extract were carried out against MRSA and MDR-*P. aeruginosa* using conventional methods to detect the minimum inhibitory concentration (MIC) and minimum bactericidal concentration (MBC) (Ekom et al., 2022). The pathogens (10^6^ CFU/mL) were inoculated into Luria Bertani (LB) broth, and the antibiofilm effect was determined using the crystal violet binding assay (O’Toole, 2011). Different extract concentrations, starting from 6.25 µg/ml up to 400 µg/ml in geometrical dilution along with bacterial culture, were added to each well of the microtire plate followed by incubation at 37 °C for 24 h. The planktonic cells were discarded, and crystal violet (20 μL) was added to the wells and allowed to stain for 15 min. The excess stain was removed, rinsed with potassium phosphate buffer (10 mM), and dried. Ethanol (96% v/v) was added to the wells to solubilize the crystal violet, and the optical density was read at 570 nm.

### 2.5 Ointment formulation and skin irritation test

The *D. anethifolia* extract formulation at two different concentrations was prepared (5% w/w and 10% w/w) employing liquid paraffin, emulsifying wax, and soft paraffin by fusion method (Nayeem et al., 2008). All the constituents of the ointment base were melted and mixed with the extract with constant stirring to obtain a uniform ointment. The physicochemical characteristics of the ointment formulation were evaluated (Kolhe et al., 2018). The formulation was applied on the mouse skin for irritation test and observed every 12 h until 72 h.

### 2.6 Antibiofilm and wound healing activity

This was done using a method standardized in our laboratory (Alrouji et al., 2023). Streptozocin and nicotinamide were used to induce diabetes (Yan, 2022). Mice were considered diabetic if the fasting blood sugar level exceeded 150 mg/dL. A coverslip containing biofilm formed by the bacteria that was confirmed by crystal violet assay (Mohamed et al., 2014) was applied to the excision wounds under anesthesia (Anesthesia (Guideline) | Vertebrate Animal Research). The biofilm formation was confirmed after 72 h by carefully removing and examining the thin biofilm layer that developed on the wounded tissue. The animals were then divided into two groups, one each for MRSA and MDR-*P. aeruginosa*, with five subgroups containing twelve animals. Group I was an untreated control, while group II was applied with the emulsifying base. The extract ointment at 5% w/w and 10% w/w was applied to animals of groups III and IV, and the last group received the local application of mupirocin 2% or gentamicin 0.1%. In six animals from each group, the wounded area was measured every 4^th^ day for 20 days, and these animals were sacrificed to determine the bacterial count (CFU/g). Tissues from these animals were also subjected to histological examination by fixing them in neutral formalin. Sections were stained using H and E stain, and skin epithelium regeneration was observed under 200X using a microscope (Leica DM 2500) with a camera (DFC 295). The epithelization period was monitored in the remaining six animals, which indicated complete healing of the wounds.

### 2.7 Cytotoxic assay on HaCaT cell lines

The SRB assay was used to determine the cytotoxicity of the extract (Denzinger et al., 2022). The HaCaT cells were grown in 96-well plates in Dulbecco’s Modified Eagle’s Medium supplemented with fetal bovine serum (10%), and antibiotic (1%) at 37°C with 5% CO_2_. Next day, extract prepared in an incomplete medium at different concentrations starting from 1 µg/ml to 1000 µg/ml was added, followed by 24 h incubation. Trichloroacetic acid - 10% (100 µl) was added, followed by incubation for another 1 h. The cells were washed in distilled water and dried, followed by the addition of sulforhodamine solution (final concentration of 0.04%) and incubation for 1 h. Following this, the cells were washed with acetic acid (1% v/v) and Tris base solution (pH=10.5) was added. This was shaken on an orbital shaker to solubilize the protein-bound dye. The optical density was read at 510 nm in an ELISA plate reader.

### 2.8 Statistical analysis

Mean ± SEM values were used for comparison, and one-way ANOVA followed by Tukey’s test was used to determine the level of significance. Instat software was used for statistical analysis (GraphPad Prism version 6.04 for Windows).

## 3 Results

### 3.1 Phytochemical analysis

**Figure 1:**
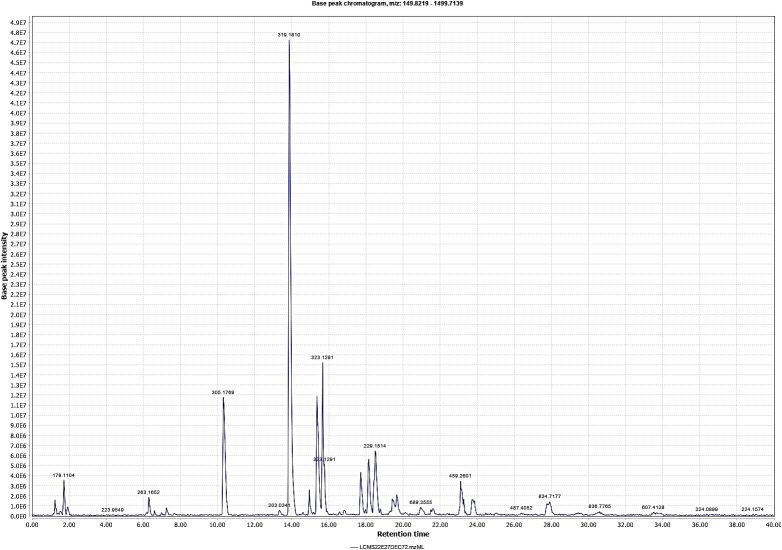
Chromatogram of *D. anethifolia* methanolic extract in positive mode. Retention times are shown in X axis and the base peak intensity of major peaks are marked.

**Figure 2:**
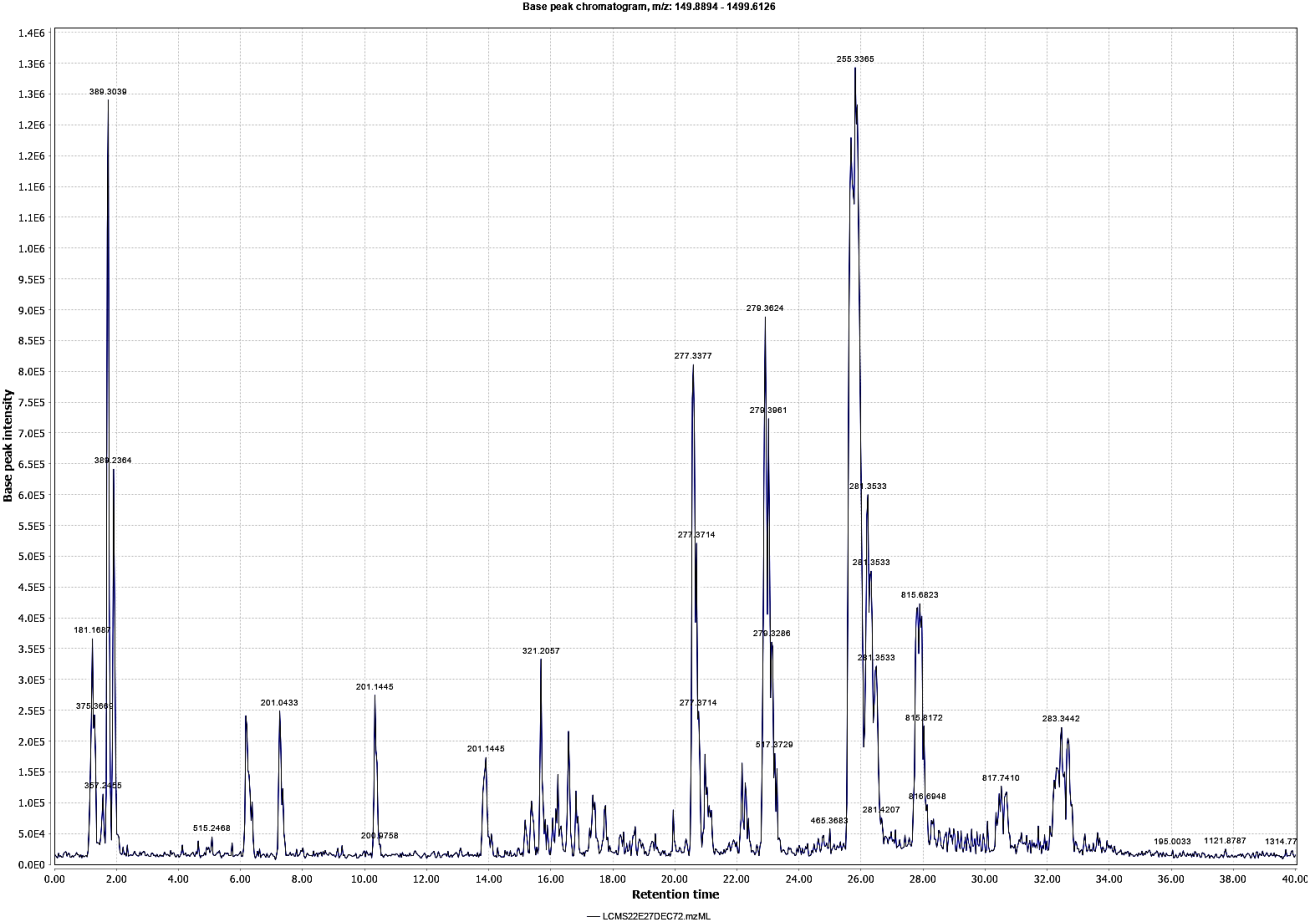
Chromatogram of *D. anethifolia* methanolic extract in negative mode. Retention times are shown in X axis and the base peak intensity of major peaks are marked.

The methanolic extract of *D. anethifolia* showed the presence of a large number of phytoconstituents in LC-MS analysis (**Figures 1**, **2**). In the positive (**Table 1**) and negative (**Table 2**) modes, 14 and 37 suspected molecules were identified, respectively.

**Table 1:** List of suspected molecules identified in *D. anethifolia* methanolic extract in positive mode.

S.No.	R.Time	Score	Compound Name	Formula	Exact Mass	Observed Mass	Mass Diff
1.	1.27	0.978	1,10-Phenanthroline monohydrate	C_12_H_8_N_2_	180.068	179.1104	0.96
2.	6.29	0.957	Adenosine	C_10_H_13_N_5_O_4_	267.096	263.1652	3.93
3.	10.32	0.979	D-erythro-Dihydrosphingosine	C_18_H_39_NO_2_	301.298	305.1769	-3.88
4.	14.95	0.935	Scoulerin	C_19_H_21_NO_4_	327.147	323.1966	3.95
5.	15.36	0.928	Methyl Jasmonate	C_13_H_20_O_3_	224.141	224.1574	-0.02
6.	17.72	0.934	DL-Dihydrozeatin	C_10_H_15_N_5_O	221.127	224.1236	-3
7.	18.16	0.978	Etidronic acid	C_2_H_8_O_7_P_2_	205.974	203.0679	2.91
8.	18.50	0.975	L-Carnosine	C_9_H_14_N_4_O_3_	226.23	229.1514	-2.92
9.	19.42	0.592	1-Isothiocyanato-8-(methylsulfinyl)-octane	C_10_H_19_NOS_2_	233.09	235.1915	-2.1
10.	19.66	0.676	Melatonin	C_13_H_16_N_2_O_2_	232.121	235.2590	-3.14
11.	23.10	0.902	Riboflavin-5′-monophosphate sodium salt hydrate	C_17_H_21_N_4_O_9_P	456.104	459.2601	-3.16
12.	23.17	0.887	peonidin-3-o-beta-d-glucopyranoside	C_22_H_23_O_11_	463.124	459.3276	3.8
13.	23.72	0.685	Hydroxypyruvic acid dimethyl ketal phosphate tri(cyclohexylammonium) salt	C_5_H_11_O_8_P	230.019	329.2370	-99.22
14.	27.91	0.767	n-Butyryl coenzyme A lithium salt hydrate	C_25_H_42_N_7_O_17_P_3_S	837.157	834.7177	2.44

**Table 2:** List of suspected molecules identified in *D. anethifolia* methanolic extract in negative mode.

S.No.	R.Time	Score	Compound Name	Formula	Exact Mass	Observed Mass	Mass Diff
1.	1.23	0.757	D(-)-Gulono-gamma-lactone	C_6_H_10_O_6_	178.047	181.1687```	178.05
2.	1.30	0.467	Galactinol Dihydrate	C_12_H_22_O_11_	342.116	375.3669	-33.25
3.	1.57	0.752	Chlorogenic acid Hemihydrate	C_16_H_18_O_9_	354.095	357.2455	-3.15
4.	1.91	0.938	R-2-hydroxy-3-butenyl glucosinolate (progoitrin)	C_11_H_19_NO_10_S_2_	389.045	389.2364	-0.19
5.	6.17	0.926	Lignoceric Acid	C_24_H_48_O_2_	368.365	367.2005	1.16
6.	6.38	0.914	Gluconasturtiin	C_15_H_21_NO_9_S_2_	423.065	423.1850	-0.12
7.	7.26	0.976	Sebacic acid	C_10_H_18_O_4_	202.12	201.0433	1.08
8.	7.37	0.799	6-(gamma,gamma-Dimethylallylamino)purine	C_10_H_13_N_5_	203.117	201.1445	1.97
9.	10.33	0.694	S-Sulfocysteine	C_3_H_7_NO_5_S_2_	200.976	201.1445	-0.17
10.	13.91	0.759	DL-4-Hydroxy-3-methoxymandelic acid	C_9_H_10_O_5_	198.052	201.1445	-3.09
11.	15.38	0.678	Petunidin	C_16_H_13_O_7_	317.066	315.1653	1.9
12.	15.69	0.658	zearalenone	C_18_H_22_O_5_	318.146	321.2057	-3.06
13.	16.23	0.93	Kaempferol-3-O-alpha-L-rhamnoside	C_21_H_20_O_10_	432.105	433.4439	-1.34
14.	16.81	0.804	Sodium Cholate Hydrate	C_24_H_40_O_5_	408.57	409.3091	-0.74
15.	17.36	0.816	Sodium gluconate	C_6_H_12_O_7_	196.058	199.1874	-3.13
16.	17.43	0.783	Syringic Acid	C_9_H_10_O_5_	198.052	199.1874	-1.14
17.	17.77	0.739	Pyridoxal-5’-phosphate hydrate	C_8_H_10_NO_6_P	247.024	249.3301	-2.31
18.	19.95	0.679	Uridine-5′-diphosphoglucuronic acid trisodium salt	C_15_H_22_N_2_O_18_P_2_	580.034	579.5029	0.53
19.	20.60	0.988	6-Phosphogluconic acid Barium salt hydrate	C_6_H_13_O_10_P	276.024	277.3377	-1.31
20.	20.70	0.894	Phloridzin	C_21_H_24_O_10_	436.136	277.3714	158.76
21.	20.77	0.957	L-saccharopine	C_11_H_20_N_2_O_6_	276.132	277.3714	-1.24
22.	20.97	0.882	2’-Deoxycytidine	C_9_H_13_N_3_O_4_	227.09	277.2955	-50.21
23.	21.04	0.876	L-Carnosine	C_9_H_14_N_4_O_3_	226.106	227.3293	-1.22
24.	21.11	0.801	Sinapic acid	C_11_H_12_O_5_	224.068	227.2618	-3.19
25.	22.17	0.892	D-Glucosamine-6-phosphate sodium salt	C_6_H_14_NO_8_P	259.045	253.3457	5.7
26.	22.92	0.976	6-Phosphogluconic acid Barium salt hydrate	C_6_H_13_O_10_P	276.024	279.3624	-3.34
27.	23.02	0.957	gamma-Linolenic acid	C_18_H_30_O_2_	278.43	279.3961	-0.97
28.	23.12	0.735	acacetin	C_16_H_12_O_5_	284.068	279.3286	4.74
29.	23.22	0.694	Guanosine-5’-triphosphate sodium salt	C_10_H_16_N_5_O_14_P_3_	522.99	517.3729	5.62
30.	23.29	0.673	Piperacillin sodium salt	C_23_H_27_N_5_O_7_S	517.163	517.3391	-0.18
31.	25.68	0.975	alpha-D-glucose-1-phosphate dipotassium salt dihydate	C_6_H_13_O_9_P	260.029	255.4040	4.63
32.	25.81	0.982	D-Glucose-6-phosphate sodium salt	C_6_H_13_O_9_P	260.029	260.03	0.001
33.	25.88	0.966	D-Mannose-6-phosphate barium salt hydrate	C_6_H_13_O_9_P	260.029	255.3703	4.66
34.	26.22	0.911	Luteolin	C_15_H_10_O_6_	286.047	281.3533	4.69
35.	26.50	0.96	Xanthosine	C_10_H_12_N_4_O_6_	284.075	281.3533	2.72
36.	27.83	0.566	Glycyrrhizin	C_42_H_62_O_16_	822.403	815.6823	6.72
37.	27.89	0.485	Glycyrrhizic acid ammonium salt	C_42_H_62_O_16_	822.403	815.6823	6.72

### 3.2 Antibacterial and antibiofilm activity

**Figure 3:**
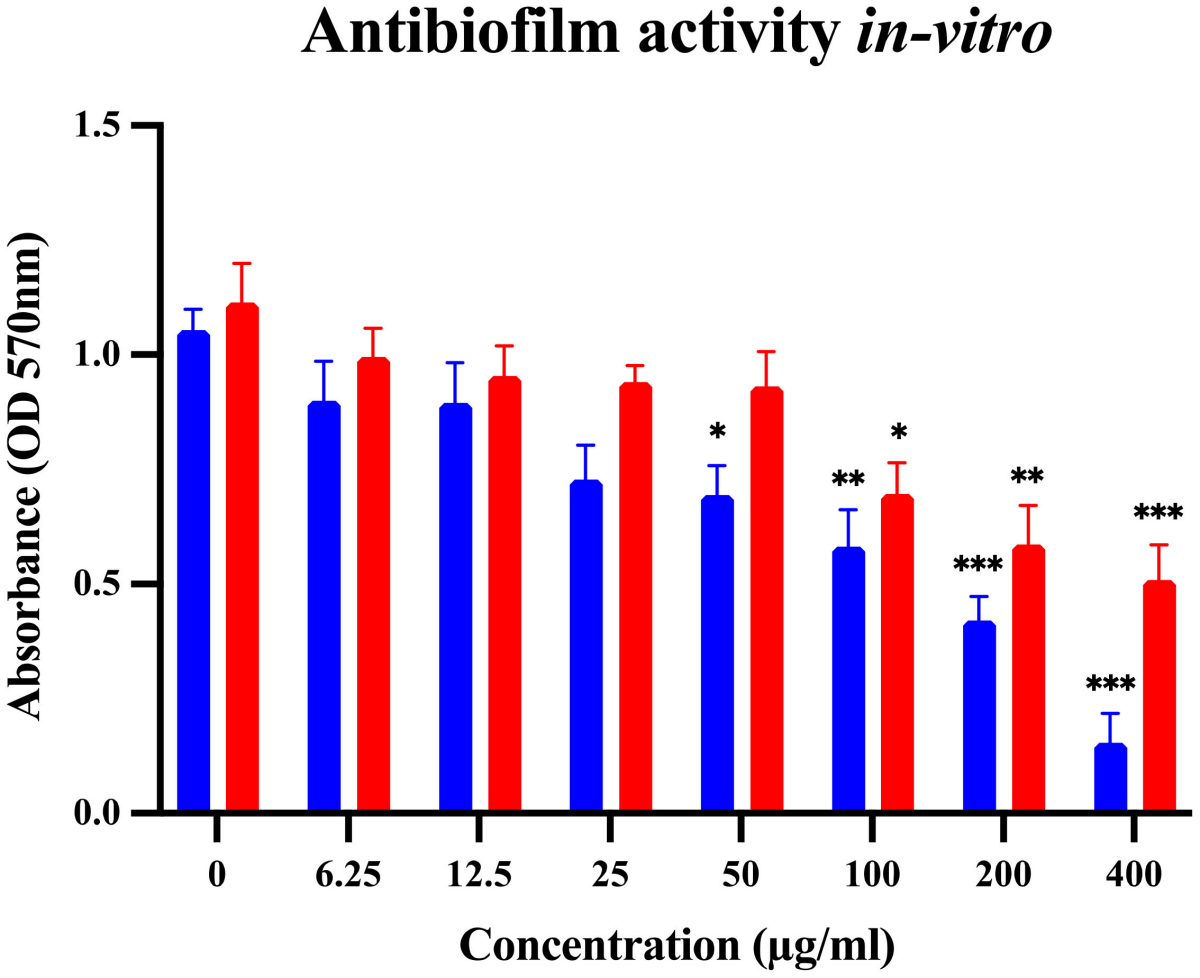
Antibiofilm activity of *D. anethifolia* methanolic extract in crystal violet assay, n= 4 (biological repeats) *P<0.05, ** P0.01, ***P<0.001 as compared to untreated control. A concentration-dependent anti-biofilm effect was observed. Activity against Gram-positive MRSA was noticeably more compared to that observed against Gram-negative MDR-*P. aeruginosa*.

The minimum inhibitory concentration was 256 µg/ml for MRSA and 512 µg/ml for MDR-*P. aeruginosa*. The minimum bactericidal concentration was 512 µg/ml for MRSA and 1024 µg/ml for MDR-*P. aeruginosa.* A concentration of 50 µg/ml exhibited significant antibiofilm activity against MRSA while MDR-*P. aeruginosa* biofilm formation was significantly affected at 100 µg/ml, and these effects were concentration-dependent (**Figure 3**).

### 3.3 Physicochemical properties and skin irritation test

The ointment formulation was homogenous with excellent stability and diffusion. The spreadability was 10 seconds with a diffusion of 0.6 cm. The prepared ointment was stable at 24°C, 37°C and 40°C. Extract formulation, when applied on intact skin, showed no obvious irritation or inflammation for 72 h.

### 3.4 Antibiofilm and wound healing effects

**Figure 4:**
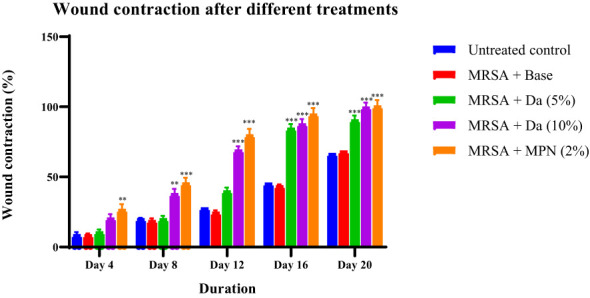
Contraction of excision wound infected with MRSA in mice treated with *D. anethifolia.* methanolic extract. The extract (Da -10% w/w) showed effect from Day 8 onwards while the mupirocin (MPN) was more effective than either concentration of the extract. All values are mean ± SEM for six animals, ^**^P<0.01, ^***^P<0.001 as compared to untreated infected control.

**Figure 5:**
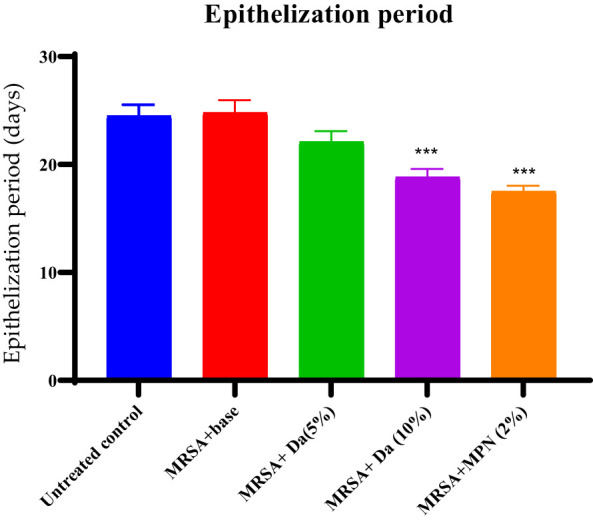
Period of epithelisation in excision wound infected with MRSA after different treatments. The extract (Da -10% w/w) and mupirocin (MPN-2% w/w) showed significant action on wound contraction while extract at lower concentration (Da -5% w/w) did not show significant effect. All values are mean ± SEM, n=6, ^***^P<0.001 as compared to untreated infected control.

**Figure 6:**
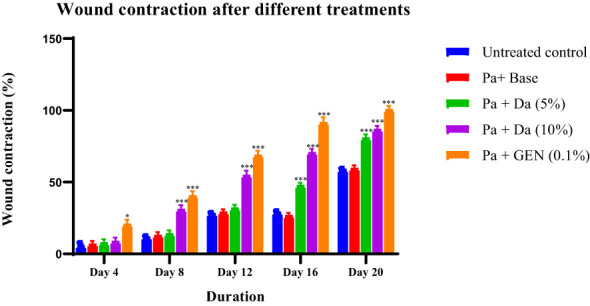
Contraction of excision wound infected with *P. aeruginosa* in mice treated with *D. anethifolia* methanolic extract and gentamicin. The extract (Da -10% w/w) showed effect from Day 8 onwards while the gentamicin (GEN) was more effective than either concentration of the extract showing significant effect from Day 4 onwards. All values are mean ± SEM, n=6, ^*^P<0.05, ^***^P<0.001 as compared to untreated infected control.

**Figure 7:**
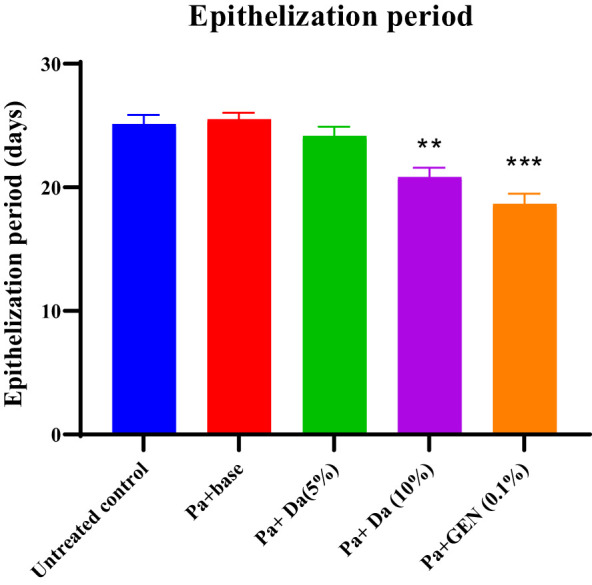
Period of epithelisation in excision wound infected with *P. aeruginosa* after treatment with *D. anethifolia* extract and gentamicin. The extract (Da -10% w/w) and gentamicin (GEN-0.1% w/w) showed significant effect on wound contraction while extract at lower concentration (Da -5% w/w) did not show significant effect. All values are mean ± SEM, n=6, ^**^P<0.01, ^***^P<0.001 as compared to untreated infected control.

**Figure 8:**
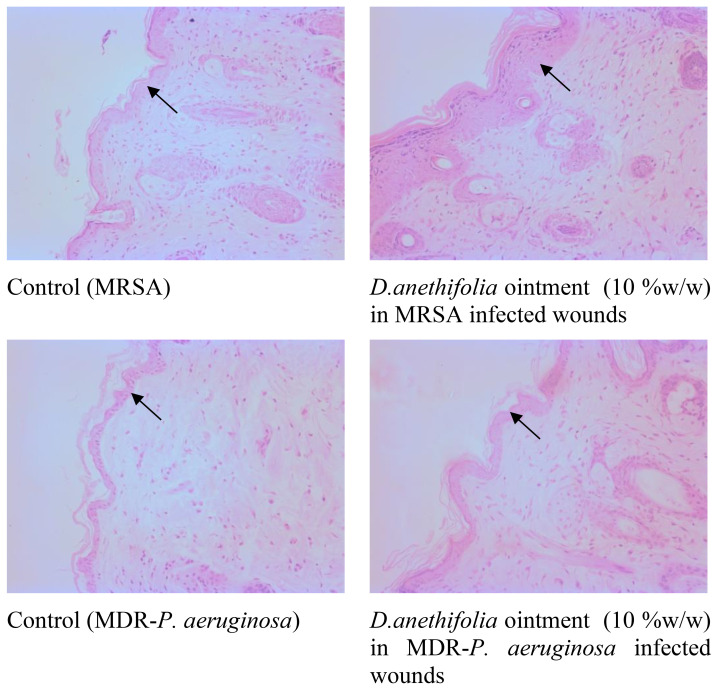
Representative images of skin section after treatment with higher concentrations of *D. anethifolia* extract (H and E stained, 200 X). In the control animals, the skin epithelial width is less when compared to the treated animals (arrow indicates skin epithelium).

The *D. anethifolia* ointment formation improved the healing of wounds in diabetic mice. The extract formulation (10% w/w) significantly supported wound healing from the 8^th^ day onwards in MRSA-induced biofilm wounds. However, the lower concentration of the extract formulation (5% w/w) showed a significant wound-healing effect from the 12^th^ day. The antibiotic mupirocin significantly affected wound contraction from the 4^th^ day. There was no significant difference in the infected wound in animals that did not receive any treatment and the base-treated wounds, indicating that the base is inert (**Figure 4**). The epithelization period was significantly reduced in low (5% w/w) and high (10% w/w) concentration extract-treated groups compared to the control. As expected, the epithelization period was significantly less in the antibiotic-treated group than in the base-treated control group (**Figure 5**). These effects were similar in MDR-*P. aeruginosa* induced biofilm wounds, but the effect of the extract was noticeably less than that observed with MRSA-infected wounds (**Figures 6**, **7**). The microbial load in the wounded tissue after 20 days of treatment was reduced after treatment with both concentrations of *D. anethifolia* extract ointment in case of MRSA-infected wounds. However, in MDR-*P. aeruginosa* infected wounds, there was a significant decrease only in wounds treated with the high concentration of *D. anethifolia* extract ointment (10% w/w). Antibiotic treatments significantly reduced the microbial load in the wounded tissue (**Table 3**). Skin sections obtained from animals receiving different treatments showed various degrees of skin regeneration. The skin damage was more in the MDR-*P. aeruginosa* infected control animals compared to MRSA-infected control animals, indicating severe skin damage due to Gram-negative MDR-*P. aeruginosa* as compared to Gram-positive MRSA. Similarly, skin regeneration after treatment with antibiotic or *D. anethifolia* extract ointment was noticeably more in MRSA-infected animals than MDR-*P. aeruginosa* infected animals (**Figure 8**).

**Table 3:** Microbial load in the wounded tissue after different treatments for 20 days in infected mice.

Group	Log_10_ CFU/g of tissue	
	MRSA	*P. aeruginosa*
Untreated control	5.23 ± 0.054	5.38 ± 0.063
Control (base)	5.12 ± 0.086	5.23 ± 0.082
*D.anethifolia* ointment (5%w/w)	3.25 ± 0.092^***^	5.09 ± 0.085^NS^
*D.anethifolia* ointment (10%w/w)	2.04 ± 0.024^***^	4.78 ± 0.092^**^
^#^Antibiotic	1.24 ± 0.046^***^	1.82 ± 0.054^***^

^#^Antibiotic-mupirocin (2%) for the MRSA-infected group and gentamicin (0.1%) for *P.* aeruginosa-infected group. Data are mean ± SEM, n=6,^**^P<0.01;^***^P<0.001 in comparison to the control (base); ^NS^Non significant.

### 3.5 Effect on HaCaT cells *in-vitro*

**Figure 9:**
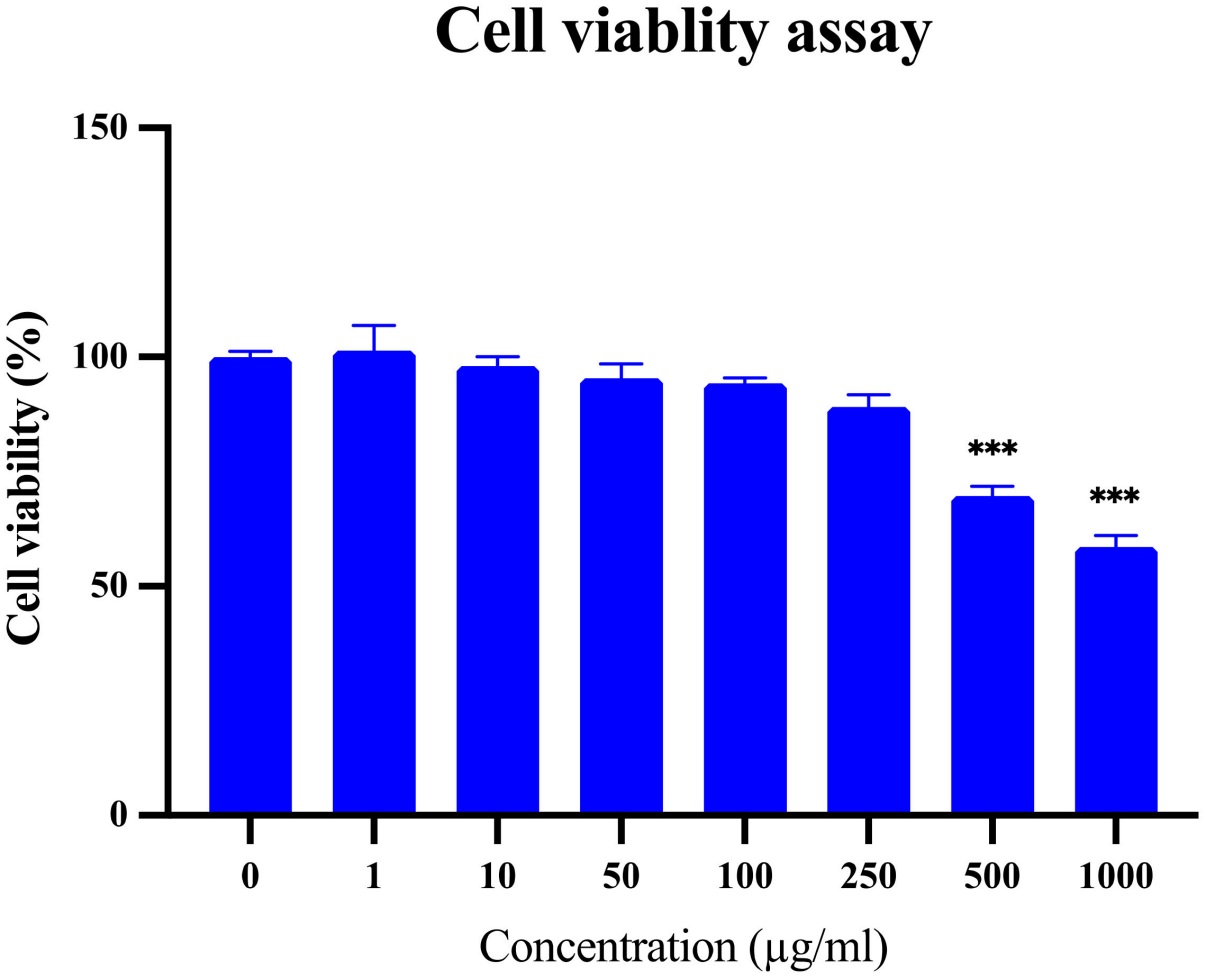
Cell viability of HaCaT cells after treatment with different concentrations of *D. anethifolia* extract in SRB assay, n=4, ***P<0.001 as compared to untreated control. There was no cytotoxic effect up to concentrations of 100 µg/ml and the IC_50_ value was 1381 µg/ml.

*Ducrosia anethifolia* did not induce significant toxicity to the HaCaT cell lines *in-vitro*, as indicated by a high IC_50_ value of 1381 µg/ml (**Figure 9**). The extract was tested up to a concentration of 1000 µg/ml, and a significant reduction in cell viability was observed at 500 µg/ml.

## 4 Discussion

*Ducrosia anethifolia* is traditionally used in different regions of the world for the treatment of skin infections and pain relief (Mottaghipisheh et al., 2020). The current study was undertaken because this herb is widely used in Saudi folk medicine to treat skin infections. The results of the current study supported its traditional use as indicated by its antimicrobial, antibiofilm, and wound-healing effects. Though there are earlier reports on the antimicrobial effect of *Ducrosia anethifolia*, none of these studies determined the antibiofilm activity and wound healing effect (Mottaghipisheh et al., 2020). The traditional use of this herb in skin infection may not be due only to antimicrobial effects, without considerable antibiofilm and wound healing properties, which were confirmed in the current study.

*Ducrosia anethifolia* extract was prepared using methanol that extracts several secondary and primary metabolites (Jones et al., 2006). Analysis of the prepared extract using LC-MS revealed the presence of many constituents. Some of the suspected phytoconstituents identified in the current study have been reported earlier for antimicrobial and antibiofilm effects. These phytoconstituents include D-erythro-dihydrosphingosine, petunidin, L-carnosine, and melatonin. D-erythro-dihydrosphingosine is a sphingolipid that has been reported to inhibit the growth of several strains of bacteria by increasing the permeability of the bacterial cell membrane (Shin et al., 2022). Petunidin, an anthocyanidin flavonoid, has a good antioxidant effect. It is also reported for antibacterial effects (Jeyaraj et al., 2022). These effects help in wound healing. L-carnosine, a dipeptide composed of amino acids, β-alanine, and histidine have been reported for antioxidant, anti-inflammatory, and antibacterial actions (Kandhasamy et al., 2021). The anti-inflammatory effect may have increased the healing of wounded tissue with the contribution of antioxidant and antibacterial actions that inhibited oxidative stress and microbial load, respectively. Melatonin is a hormone found in both animals and plants. It has potent antioxidant, anti-inflammatory, and immunomodulatory properties that aid in the healing of wounds (Ganganna et al., 2021).

Another important phytoconstituent identified in the plant was chlorogenic acid. It is a polyphenol found in several plants, including vegetables and fruits. There are several reports on the antibacterial effect of chlorogenic acid, and it is reported to inhibit several strains of bacteria, confirming its broad-spectrum antibacterial action (Sun et al., 2020). The extract also showed the presence of kaempferol, which is a known antibacterial agent. It is reported to increase cell membrane permeability, inhibit bacterial enzyme activity, and have a strong antioxidant effect (Periferakis et al., 2022). Similar to kaempferol, syringic acid, and sinapic acid is found in several plant species, and these are known to inhibit bacterial growth by a mechanism similar to kaempferol (Pandi and Kalappan, 2021; Meng et al., 2022). Sodium gluconate is abundantly found in several plants. It is a chelating agent that chelates ions essential for bacterial growth, and there are few reports on the antibacterial effect of this compound (Kapanya et al., 2020). Luteolin is an important flavonoid that is reported for antibacterial activity against a wide range of both gram-positive and gram-negative bacteria. It also has anti-inflammatory and antioxidant properties that help in wound healing (Guo et al., 2020). Glycyrrhizin, commonly found in licorice, was found to be present in *D. anethifolia*. There are many reports on the broad-spectrum antibacterial effect of glycyrrhizin (Eynde et al., 2023). It also possesses antioxidant, anti-inflammatory, and immunomodulatory effects (Feng et al., 2022). Furthermore, glycyrrhizin has been reported to enhance the antibacterial effects of many conventionally used antimicrobial agents (Hazlett et al., 2019). The presence of various phytoconstituents with diverse pharmacological effects that include antioxidant, anti-inflammatory, immunomodulatory, and antibacterial effects might have contributed to the overall observed effects.

The phytochemical analysis of *D. anethifolia* has been carried out by several authors in different extracts prepared using different solvents such as aqueous, ethanol, and ethyl acetate. A comparison of the phytoconstituents reported by these authors with those found in this study did not match any of the constituents (Zamyad et al., 2019; Mottaghipisheh et al., 2020; Arabsalehi et al., 2022). The reason for this cannot be explained by the present data. However, this could be due to the place and time of collection of the plant material and method of analysis, as some of these studies were carried out using gas chromatography-mass spectrometry (GC-MS). Many reports are from Iran, which has different weather conditions than Saudi Arabia. Further, the current study was carried out using methanol extract, and there are no earlier reports on the phytochemicals present in the methanolic extract of *D. anethifolia*.

Many plants have been reported for antibacterial and wound healing effects in normal and diabetic rats (Al-Ghanayem et al., 2022b; Almuhanna et al., 2023). However, there are very few reports on the *in-vivo* antibiofilm effects of plants and phytoconstituents (Lu et al., 2019). Several plant-based formulations are reported to control infection in diabetic wounds but their efficacy on biofilm is unknown. To overcome antimicrobial resistance, environmental degradation, and pollution, plant-based formulations are becoming safer alternatives for antibiotics and have gained importance in recent. Apart from antibacterial activity, many of the plant components are reported for enhancing fibroblast proliferation, a main step in wound healing (Thakur et al., 2011). In Middle East traditional plants including *Ducrosia anethifolia* are used as a traditional medicine.

Management of wounds in diabetic conditions is a serious concern as pathogens such as MRSA and MDR-*P. aeruginosa* are resistant to conventionally used antibiotics. Both these pathogens were selected based on literature and as a representative strain from Gram-positive bacteria and Gram-negative bacteria to establish the wide spectrum of activity. The extract showed a more antibacterial effect on MRSA when compared to MDR-*P. aeruginosa*. Usually, Gram-negative bacteria are more tolerant to phytochemicals and natural compounds compared to Gram-positive bacteria due to the different physiological structures of the cell walls. The lipopolysaccharide layer and periplasmic space of the cell wall help the Gram-negative bacteria to show resistance against natural compounds (Al-Ghanayem et al., 2022b).

Treatment of biofilm-formed wounds requires the use of strong antimicrobials and proper care, and in a few cases, surgery may be required (Ruhal and Kataria, 2021). Herbs and phytochemicals have been reported for antibiofilm and wound-healing properties. This includes *Aloe vera*, curcumin, allicin, and many essential oils. It is believed that herbs and phytochemicals may hold promising benefits in the management of biofilm infections and wound care (Karygianni et al., 2016).

In the current study, biofilms were induced on excision wound in diabetic animals. Wounds in diabetic condition provide a suitable environment for the formation of biofilms, and if untreated, it may lead to gangrene. There are several animal models for the development of biofilm. The method adopted in this study was developed and validated in our laboratory (Alrouji et al., 2023). The selection of two different concentrations was based on pilot studies and skin irritation studies. There are several studies on different plant extracts using the same concentrations (Taddese et al., 2021; Tekleyes et al., 2021). The ointment in a suitable base was used to increase the stability, spreadability, and diffusion (Kolhe et al., 2018). The MIC of the extract was 256 µg/ml for MRSA and 512 µg/ml for MDR-*P. aeruginosa*, which shows that the pathogens are precisely inhibited at different concentrations. These values are higher compared to conventionally used antibiotics that are pure chemicals. The MIC values are always higher for crude extracts that contain several phytoconstituents as compared to pure chemicals and isolated phytoconstituents. Isolation of active constituents from this crude extract may lead to new lead molecules having potent antibacterial effects.

The present study is on crude methanol extracts of *Ducrosia anethifolia.* Identifying potential phytochemicals possessing antibacterial and antibiofilm effects may further help to explore novel compounds for treating MRSA or MDR- *P. aeruginosa*-infected diabetic wounds. The wounds were infected with single pathogens, either MRSA or MDR- *P. aeruginosa*; however, in diabetic wounds, polymicrobial infections and biofilms were also formed. Further studies on polymicrobial antibiofilm activity and infection control may provide in-depth knowledge on the efficacy of the *Ducrosia anethifolia* extract. The study conducted was focused on the excision wound model. Extending the studies on different wound models may also provide insight into the wound-healing properties of the extract.

This study determined antibacterial, antibiofilm and wound healing properties of the crude methanolic extract of *Ducrosia anethifolia*. There can be multiple mechanisms for wound healing action of the plant extract apart from antibacterial and antibiofilm effects. These include cell proliferative actions, and antioxidant effects. There are reports on the antioxidant effect of *D. anethifolia* but its effect on cell proliferation in the skin is unknown (Elsharkawy et al., 2019).

Though this study determined both *in-vivo* and *in-vitro* antibiofilm activity of *D. anethifolia* extract, it has a few limitations. The present work determined the activity of the crude extract of the plant and the contribution of each phytoconstituent present in the extract to the observed effects was not assessed. This is important to determine the synergistic and antagonistic effects of the combination of phytoconstituents, as earlier reports on *D. anethifolia* showed that volatile oils are effective antimicrobial agents while its main phytoconstituent-decanal was less effective suggesting synergistic effects of different molecules present in the extract (Mahboubi and Feizabadi, 2009). The present study was done using only one model of wound healing. Effect on other models of wound healing such as the incision-wound model, and burn-wound model may help to substantiate the effect of *D. anethifolia* on the wound healing process (Sami et al., 2019).

## 5 Conclusion

The methanolic extract of *Ducrosia anethifolia* showed good antibacterial, antibiofilm, and wound healing properties. The antibacterial effect was dose-dependent, and the effect was more against MRSA than MDR-*P. aeruginosa*. The extract did not produce any skin irritation and was also safe on HaCaT cell lines. The LC-MS analysis of the extract revealed the presence of several phytochemicals, some of which have been reported for antibacterial, antioxidant, and anti-inflammatory actions. The effects observed in the current study could be due to multiple phytoconstituents, and evaluating individual bioactive phytoconstituents may help in the discovery of novel antibacterial and antibiofilm agent(s). The results of the study may help in identifying novel molecules that may positively affect the different phases of the wound healing process.

## Data availability statement

The raw data supporting the conclusions of this article will be made available by the authors, without undue reservation.

## Ethics statement

The animal study was approved by Ethical Research Committee Shaqra University. The study was conducted in accordance with the local legislation and institutional requirements.

## Author contributions

YA: Writing – review & editing, Writing – original draft, Visualization, Validation, Supervision, Software, Resources, Project administration, Methodology, Investigation, Funding acquisition, Formal analysis, Data curation, Conceptualization.
